# Parental Attitudes toward Consent for Music Intervention Studies in Preterm Infants: A Cross-Sectional Study

**DOI:** 10.3390/ijerph18157989

**Published:** 2021-07-28

**Authors:** Sofia Bauer, Shulamit Epstein, Łucja Bieleninik, Dana Yakobson, Cochavit Elefant, Shmuel Arnon

**Affiliations:** 1 Department of Neonatology, Meir Medical Center, Kfar Saba 44281, Israel; Bauers@clalit.org.il (S.B.); Dandajacob@gmail.com (D.Y.); 2 Sackler Faculty of Medicine, Tel Aviv University, Tel Aviv 39040, Israel; 3 School for Creative Arts Therapies, University of Haifa, Haifa 3498838, Israel; shushuq@gmail.com (S.E.); celefant@univ.haifa.ac.il (C.E.); 4 Faculty of Social Sciences, Institute of Psychology, University of Gdańsk, 80-309 Gdansk, Poland; Lucja.Bieleninik@ug.edu.pl; 5 GAMUT—The Grieg Academy Music Therapy Research Centre, NORCE Norwegian Research Centre AS, 5029 Bergen, Norway

**Keywords:** observational study, music therapy, parental consent, preterm infants, survey

## Abstract

(1) Background: This study investigated parents’ motives for enrolling preterm infants into music therapy intervention studies during Neonatal Intensive Care hospitalization. (2) Methods: We surveyed Israeli parents of preterm infants after they consented or refused to participate in such studies. The pre-piloted questionnaires evaluated attitudes toward research and music therapy intervention studies. The study included 116 (57%) parents who agreed to participate in music therapy studies and 87 (43%) who declined. (3) Results: Infants of those who agreed to participate were younger (17 ± 2.3 vs. 28 ± 4.7 days old, *p* = 0.03) and sicker (Clinical Risk Index for Babies score 6.1 ± 2.7 vs. 3.68 ± 4.1, *p* = 0.04). More single-parent families declined to participate (*p* = 0.05). Parents agreed to participate because they thought the study might help their child, would improve future care of preterm infants and increase medical knowledge (all *p* < 0.05). In addition, they perceived music as beneficial for brain development, thought it might improve bonding, and routinely listened to music daily. (4) Conclusions: When recruiting parents and preterm infants for music therapy intervention studies, one should highlight potential contributions to the child’s health, future children’s health and medical knowledge. Stressing music as a potential tool for brain development and augmenting bonding is important. The best time to recruit is when improvements are still anticipated.

## 1. Introduction

The opportunities for advancements in the prevention and treatment of diseases that affect preterm infants are crucial to improving care for this vulnerable population [1]. Advances in clinical medicine are often the result of clinical research; however, studies on preterm infants are limited, due to the extra care required when designing studies with this population. In addition, it is difficult for parents to decide whether to consent in these circumstances [2]. To the best of our knowledge, the views and perceptions of parents about the benefits of research and their participation as proxy consent givers have been assessed in only a few studies so far [2,3,4,5,6,7,8]. The methodology of studies that explored parental willingness to participate in research during their infants stay in the Neonatal Intensive Care Unit (NICU) included questionnaires [2], surveys [3,4,6], systematic reviews [8] and qualitative analyses [5]. As characterized in a few studies, the primary motives of parents to participate in clinical trials during their children’s NICU hospitalization were helping other infants, families or physicians; increasing the possibility of helping their own baby or themselves; and a general perception that trials engender hope [2,8]. Reasons for declining to participate in studies were documented as unwillingness of parents to burden their child; worries about risk-factors; urgency of decision making; and creating pressure and inconvenience for the parents in their stressful situation [6,8]. In addition, parents are more likely to consent to studies that impose fewer risks to their baby [2,6]. Therefore, caregivers tend to agree to participate in psychological, as opposed to pharmaceutical or vaccine-based studies [2,4]. A balancing factor for participating in higher risk studies is the extent of perceived benefit. Parents of infants in the NICU focus on understanding the clinical procedures and potential risks of the study when consenting to participate. Once they understood the benefits, parental consent was not affected by the severity of their baby’s illness [7,8].

Comprehensive studies involving music therapy (MT) interventions showed benefits in a few domains, which increased interest from the medical and parental community [9,10,11,12,13,14,15]. Creative music therapy, an individualized, interactive, resource and needs-oriented approach, was shown to have beneficial effects on functional brain activity and connectivity in networks underlying higher-order cognitive, socio-emotional, and motor functions in preterm infants [13]. The willingness of parents to participate with their preterm infant in MT intervention studies has not been investigated in detail. It seems reasonable that parents of a vulnerable preterm infant would feel comfortable participating in studies that do not impose any threat or burden to them or to their child, such as those involving music interventions. However, this hypothesis has not been explored in depth.

The aim of this study was to collect and explore information about parental attitudes and factors regarding consent for music intervention studies during their child’s NICU hospitalization. The objectives were to determine the reasons parents report for consenting or refusing to allow their preterm infant to participate in MT intervention research while hospitalized in the NICU, whether there are demographic factors related to consenting or declining MT intervention studies, and whether there are music-related factors associated with consenting to these studies. 

We hypothesized that those who consented considered music a beneficial intervention, as compared to those who refused. Other reasons for consent were evaluated as well, and demographic characteristics were compared between those who consented and those who did not.

## 2. Materials and Methods

### 2.1. Study Design and Setting

This paper presents a cross-sectional study on parental attitudes and factors regarding consenting for music intervention studies during their child’s NICU hospitalization The study took place in the NICU at Meir Medical Center Israel from February 2016 to March 2018. The study was conducted according to the guidelines of the Declaration of Helsinki, and approved by the Institutional Review Board of Meir Medical Center (protocol number 0354-16-MMC). All participants were given written information about the study and provided signed informed consent. This 30-bed NICU serves a regional population of 600,000 people. Approximately 300 neonates are admitted to the NICU annually, of whom 60% are preterm, born before 37 weeks’ gestation. 

### 2.2. Procedures

The respondent groups were parents who had recently been approached for consent to enroll their preterm infants in one of three, independent, controlled, MT intervention studies conducted in the NICU. The three aforementioned studies were conducted during 2016–2018 (results of one was published [12]). Data were obtained by staff employed in the NICU, including a music therapist, research nurse, neonatal fellows, and neonatologists. MT intervention studies employ a certified music therapist who underwent advanced training in NICU MT prior to working with this population and their caregivers. These criteria are based on the importance of ensuring music is implemented at a safe decibel level and the infant is appropriately monitored for signs of overstimulation. 

In the current study, parents were approached 3–5 days after they consented or declined to participate in a study and were asked to complete a questionnaire about their attitude towards the MT intervention study and their attitude towards studies involving their preterm infant, in general. 

### 2.3. Respondent Group

Criteria for selecting the respondent group were as follows.

Inclusion criteria were parents who were previously approached to participate in MT-based studies, who agreed to participate in the current survey study, and their ability to understand the questionnaire. Exclusion criteria were disagreement between parents on answers or an incomplete questionnaire.

### 2.4. Variables and Data Collection

**Figure 1:**
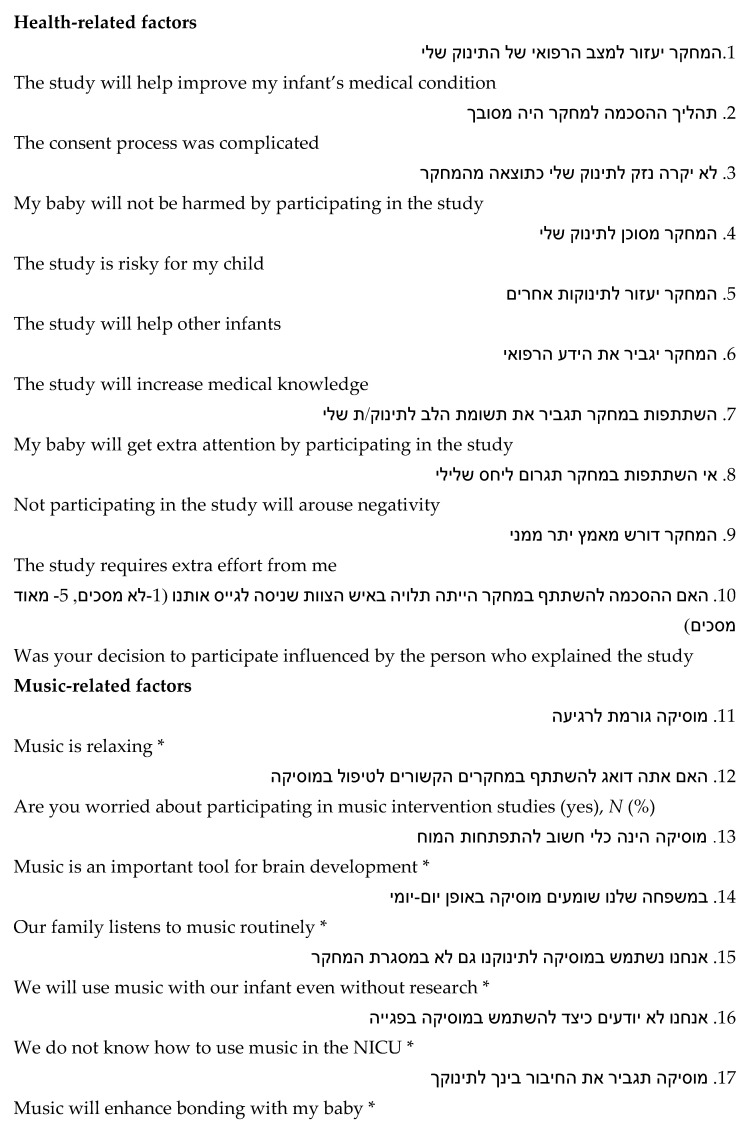
Questionnaire in the original Hebrew followed by the English translation.

A 17-item questionnaire on MT studies was developed for this study (Figure 1) to gather information on parental reasons for consenting or refusing for their preterm infant to participate in MT intervention research while hospitalized in the NICU, as well as parental attitudes towards MT dedicated to premature infants during their NICU stay. The questionnaire was written in Hebrew and included general questions about reasons to participate in a study involving preterm infants and specific questions concerning participating in MT intervention studies involving preterm infants.

Responses were based on a five-point Likert scale of: (1) Strongly disagree, (2) Disagree, (3) Neither agree nor disagree, (4) Agree, or (5) Strongly agree. Not applicable (N/A) was an optional answer. Questions assessed factors that have been suggested to affect consent. These included altruism, attitudes toward research for preterm infants in general and in the music field, their “love” for music and use of music in their daily life. Finally, one open-ended question was used to check content validity, asked for the single, most important reason for the parental decision. A copy of the anonymous questionnaire was given to the parents to be returned in a sealed envelope. Assistance with any reading difficulties was offered, but no parents availed themselves of this opportunity. The questionnaire was pretested for readability and comprehensibility on a sample of 5 parents in the NICU who were not included in the study. Face validity, as assessed by the MT intervention study personnel was good, and any misunderstood or ambiguous item was rewritten until it was clear and covered the concept of why parents involve their baby in these studies. In addition, the first 10 parents enrolled were asked to complete the questionnaire again, 2 weeks after their initial response. The intraclass correlation coefficients for the individual scaled responses ranged from *r* = 0.77 to 0.97; indicating very good test–retest reliability. The response distribution on scaled questions was good, with frequency of endorsement for at least three of five response alternatives greater than 5% on all items.

The following parental variables were collected: maternal age (years), marital status (single-parent family, yes/no), maternal and paternal education (years), employment status (yes/no), ethnic origin (Jewish, Arab or other), and self-perceived religious status (the way the parents described their daily life). Information regarding the infants included gestational age (weeks), birth weight (grams), sex (male/female), having siblings (yes/no), parents’ previous research involvement with this baby (yes/no), infant’s post-menstrual age at consent/decline (days), and severity of infant’s illness graded by Clinical Risk Index for Babies (CRIB) score [16]. CRIB score was evaluated retrospectively for the time of MT study consent or refusal.

### 2.5. Power Calculation and Data Analysis

The primary outcome was the parental assumption that music is an important tool for brain development as their motivation to enroll their preterm infant into MT intervention studies. The minimal clinically significant difference for parental assumption that music is an important tool for brain development as their motivation for enrolling their preterm infant into MT intervention studies is unknown. Therefore, the study was powered to detect a substantial effect of 25% difference in this variable. In an observational study, with a 2-sided, 5% significance level and 80% power, a sample of 181 infants was required. Continuous data are presented as mean (standard deviation, SD); all were normally distributed as assessed with the Kolmogorov–Smirnov test. Nominal variables are presented as prevalence (percentages). The two parental decisions (consent or decline) were compared using unpaired *t*-test for all continuous data. For categorical variables, chi-squared or Fisher’s exact test was used to determine significance. Data were analyzed using SPSS 15.0 (SPSS, Inc., Chicago, IL, USA), and significance was set at <0.05.

## 3. Results

**Figure 2:**
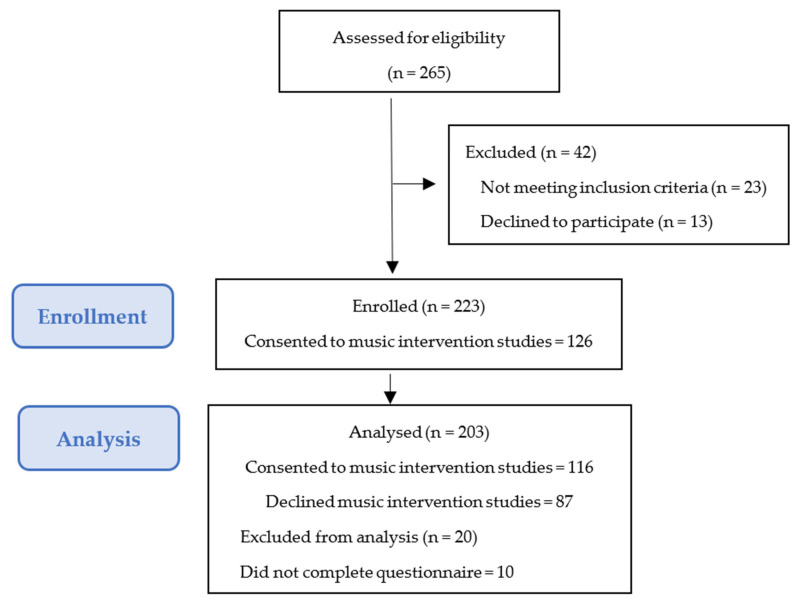
Study flow diagram.

The initial sample of those who were approached to participate in MT studies consisted of 265 families, of whom 223 (84%) completed the questionnaire on parental attitudes toward consent for music intervention studies. Among 142 (53%) who consented to MT intervention studies, 126 (89%) completed the questionnaire, and of 123 (47%) who refused to participate, 97 (79%) completed the questionnaire. No statistical difference was noted between those who consented to those who refused to participate in the MT intervention studies (*p* = 0.76). The study flow diagram is depicted in Figure 2. Twenty families were excluded from the final analysis (10 in each study group) due to incomplete questionnaires (*n* = 10) or disagreement between parents (*n* = 10).

Table 1 presents health factors related to consenting or declining to participate in MT intervention studies. Parents reported that they consented for their preterm infant to participate in MT intervention research while hospitalized in the NICU because they thought the study might help their child’s condition, improve future care of other preterm infants, and that it would increase medical knowledge (all *p* < 0.05, as compared to those who declined to participate).

Table 2 provides the results on music-related factors of those who consented or declined MT intervention studies. The significant music-related reasons for participating in studies involved MT intervention were: music is an important tool for brain development, listen to music routinely, and music will enhance bonding with the baby (all *p* < 0.05 compared to those who declined to participate).

The higher scores for the reasons reported by parents who refused to participate in MT intervention research while child’s stay at the NICU, as compared to those who agreed to participate, were related to the arguments that the consent process was complicated, the study is risky for their child, the study requires extra effort from the parent, and that refusing to consent for the study was related to the personnel who approached them for consent. Concerning the music-related factors that were associated with study refusal, no specific reason was recorded as significantly related to study refusal. However, the refusal group scored all the positive music-related factors lowers (Table 1 and Table 2).

As can be seen in Table 3, parents who agreed to participate in MT intervention studies had younger infants (17 ± 2.3 vs. 28 ± 4.7 days old, *p* = 0.03), who were considered sicker after birth (CRIB score, 6.1 ± 2.7 vs. 3.68 ± 4.1, *p* = 0.04), as compared to those who declined. There was a trend toward more single-parent families declining to participate (*p* = 0.05). No difference in parents’ education, maternal age, economic status or religious observance was found between the groups.

## 4. Discussion

To our knowledge, this is the first study to evaluate the determinants of parental decision-making regarding enrolling preterm infants in clinical MT intervention studies, examining issues regarding research among this vulnerable population and specific factors related to willingness to participate in these studies.

MT interventions have shown efficacy in addressing symptom management and developmental outcomes with preterm infants [10,11,13,14]. MT is a profession wherein music-based interventions are used by a trained music therapist to target health-related goals, using music experiences and therapeutic relationships as primary forces for positive change [14]. Preterm infants exposed to a live singing intervention by music therapists showed higher connectivity between primary auditory cortices and the thalamus, and between the prefrontal and orbitofrontal regions associated with affective and emotional processing [13]. As brain development is a primary concern of parents of preterm infants hospitalized for long periods in the NICU, our primary outcome hypothesis was that those who agreed to participate perceived music as more beneficial for brain development, which was confirmed by the study. We also found that parental agreement to consent to MT intervention studies was related to the notions that the study might help their baby’s condition, improve future care of preterm infants and increase medical knowledge. Parents who consent to MT intervention studies did not anticipate any harm to their baby, and reported using music daily in their normal life, as compared to those who declined to participate. Parents who agreed to participate in these studies felt that the study would enhance bonding with their baby. They also had younger infants who were considered sicker, as defined by the CRIB score evaluated at the time of study enrollment. This might be because parents feel that the sicker their baby is, the more benefits might be gained from MT intervention studies.

The results provide supportive evidence that research involving music intervention in the NICU might be seen by parents as beneficial for their child and for others, with positive short- and long-term implications. We speculate that similar to psychological studies, parents are more amenable to MT intervention studies as compared to pharmaceutical studies. MT intervention studies impose less risk and are more comprehensive and familiar to parents, as compared to medical intervention studies and give parents more control as the primary proxy decision-maker for their infant [4].

In agreement with other studies on parental consent, we found that parents who perceived either personal or societal benefits, were more likely to participate than if they perceived risk [2,4]. Most studies that investigated parental willingness to participate in studies involving their preterm infant, evaluated hypothetical situations, whereas our study examined an actual consent process and therefore, may be more representative.

Our results agree with those of a similar study that administered a questionnaire to parents within a few days after considering enrolling their infant in research, and reported that participation was correlated with a composite factor of “risk, benefit and attitudes” [17]. This factor included the probability and magnitude of risk and benefit, altruism, general attitude to research, perceived complexity of decisions, freedom to make decisions, and concerns about reprisal. Our study emphasizes the additional value of music as the subject of investigation during the consent process. The participants who agreed to participate perceived music as beneficial for brain development, reported using music daily in their normal life and felt that it would enhance bonding with the baby. These findings are supported by recent study trends within the MT field, showing its beneficial effects on brain development [13] and on research aiming to measure the effect of MT on parental bonding and attachment [18,19,20]. Parents view interactive and linguistic communication as very important [21] and are encouraged to read to their infant in the NICU as part of establishing better bonding and attachment [22].

Societal benefit is often cited as a reason for participating in neonatal research [2,4]. In a large European study on the informed consent process for neonatal randomized trials, 49% of the parents chose to participate to benefit future infants [23]. Our results also show that those who consent to MT intervention studies were driven by values of societal benefits as compared to those who refused.

Our results are also in agreement with a study by Hulst et al. [24], who found that illness severity scores were higher among the preterm infants for whom consent was obtained. This might be explained by parents’ perceiving more benefits from a study while their baby is ill compared to when their baby is medically stable.

Our sample is representative of the NICU population as it included a wide range of participants with heterogeneous characteristics similar to those seen by clinicians in daily practice (e.g., presence of morbidities and comorbidities). Sociodemographic variables of those who participated vs. those who declined were also similar, showing that consent for MT intervention studies was not related to demographics. Concern has been expressed that among those consenting to research there may be disproportionate representation of individuals who are unable to understand the information or who, by virtue of social disadvantage, are too intimidated to refuse [3]. Our results are reassuring, as parents who consented had similar educational levels as those who refused. We also did not find any differences in ethnic or religious characteristics between those who consented and those who declined to participate in MT intervention research.

Morley et al. [25] suggested that 22% of parents felt anxious when they were asked to participate in a clinical study. In our study, a much smaller proportion said that they were concerned (9% of those who did not consent vs. 4% of those who did), suggesting that MT intervention studies are less intimidating than medical treatment-related studies are. Care must be taken to ensure parents are not concerned about participating in research, but our results suggest that most were relatively unconcerned. One issue with questions on anxiety is that there is a high background level of parental anxiety when their baby is in a NICU. However, most parents considered music a relaxing medium, with no added anxiety to their decision.

### Strengths and Limitations

The current study had several limitations. The results cannot be generalized to the broader population because data may be associated with cultural and environmental factors associated with the Israeli society. Thus, they are limited to the cultural and social context in Israel. The MT involved in the three studies mentioned in Section 2.2. Procedures were based on employing a certified music therapist who worked together with the parents on their preferred song during skin-to-skin care with their baby. Therefore, it might be that parental decisions were influenced positively or negatively by the presence of the therapist. We did not elaborate on this, as we felt that employing a certified music therapist who underwent advanced training in NICU-MT prior to working with this population and their caregivers, due to the importance of ensuring music is implemented at a safe decibel level and the infant is appropriately monitored for signs of overstimulation, was a fundamental issue that could not be changed in the studies. Due to ease of recruiting families to complete the questionnaire among those who consented, as compared to those who refused to participate in MT intervention studies, a bias could be present. However, in our study the percentages of those who consented and refused in each group were similar (89% vs. 79%). We tried to avoid response bias (due to inability or lack of desire to answer the questionnaire) by keeping the questions short and clear, by asking questions that were discussed extensively when the family was approached for the MT intervention study and by providing options for interval responses. Another limitation was that any questionnaire-based evaluation is only an approximation of actual motives. The validity of the responses is also affected by the time lapse between the decision and questionnaire completion, because parents’ interpretation of risks may be altered by the infant’s subsequent course, and their attitudes may be affected by later experiences in the NICU. We did not find it acceptable to approach parents immediately after the consent decision. However, we approached them within few days, assuming the medical condition of these stable infants was unchanged. We were not able to determine the relative priority of parental weighing of risks and benefits and their attitudes toward research, because both loaded onto the same factor in the analysis and separating the two would require a much larger sample. Finally, psychological profiles of the parents were not determined. Parental anxiety has been correlated with consent for research [8].

Notwithstanding these limitations, a key strength of this study was that the developed questionnaire was for an actual, not hypothetical study, to understand why parents responded to study consent as they did. The questionnaire was pre-piloted based on external sample size and reached a very good test–retest reliability. Secondly, both parents participated in the study, providing mutual opinions, which resembles the actual scenario when medical staff approaches parents for consent. Third, despite its exploratory nature, the current study is based on a convenience sample of participants. Forth, the response rate (84%) was excellent which is a good indicator of survey quality and reduce the sampling bias. Fifth, caregivers who refused to participate do not differ from those who take part which minimalize non-response bias.

## 5. Conclusions

The findings of this research provide insights for parental attitudes toward consent for neonatal music intervention studies in pre-term infants and parental decision making. The following conclusions can be drawn from the present study. This study supports the notion that parents of preterm infants agree to participate in MT intervention studies when they feel it might help their child’s condition, improve future care of preterm infants and that the study would increase medical knowledge. Specifically, those who agreed to participate, perceived music as beneficial for brain development, expected MT to increase bonding with their baby and reported using music routinely in their normal life. Therefore, when recruiting parents and their preterm infants for MT intervention studies, one should highlight potential contributions of the study to the child’s health, future children’s health and medical knowledge. Emphasizing music as a potential tool for brain development and enhancing bonding are also important. The best time to recruit families is when potential improvements are still anticipated. This study has raised important questions about the nature of parental willingness to participate in clinical trials. The results obtained in this study are relevant to investigators recruiting patients for future MT studies in the NICU. However, further research that tries to understand why parents agree or decline to participate in studies concerning preterm infants should be extended to other allied health groups. Characterizing studies that are understandable for parents either by their subject or by the written consent explained by the research team, studies that give parents hope for improving their child’s prognosis, along with parental sense of responsibility might enhance their willingness to participate.

## Figures and Tables

**Table 1:** Health factors related to consenting or declining to participate in music therapy intervention studies.

Health-Related Factor	Consented (*n* = 126) Mean ± SD	Declined (*n* = 97) Mean ± SD	*p*-Value
The study will help improve the infant’s medical condition	4.28 ± 0.62	2.17 ± 0.85	0.03
Consent process was complicated	2.35 ± 1.1	2.85 ± 1.3	0.27
My baby will not be harmed by participating in the study	3.54 ± 0.45	2.95 ± 0.72	0.16
The study is risky for my child	1.34 ± 0.72	2.21 ± 1.3	0.37
The study will help other infants	3.92 ± 0.72	2.26 ± 0.41	0.04
The study will increase medical knowledge	4.44 ± 0.75	2.2 ± 0.56	0.01
My baby will get extra attention by participating in the study	2.73 ± 1.2	2.51 ± 1.3	0.23
Not participating will arouse negativity	2.7 ± 0.32	2.16 ± 0.47	0.24
The study requires extra effort from me	2.1 ± 1.3	2.5 ± 1.8	0.32
Was your decision influenced by the person who explained the study (1—No, 5—Yes)	3.2 ± 1.2	3.5 ± 0.8	0.6

Annotation: SD, Standard Deviation; for the answer categories: (1) Strongly disagree, (2) Disagree, (3) Neither agree nor disagree, (4) Agree, (5) Strongly agree.

**Table 2:** Music-related factors of those who consented or declined music therapy intervention studies.

Music-Related Factor	Consented (*n* = 116)	Declined (*n* = 87)	*p*-Value
Music is relaxing *	3.8 ± 1.2	2.9 ± 1.5	0.24
Are you worried about participating in music intervention studies (yes), *n* (%)	5 (4)	8 (9)	0.26
Music is an important tool for brain development *	4.66 ± 0.58	2.74 ± 1.2	0.03
Our family listens to music daily *	3.8 ± 0.81	1.6 ± 0.49	0.03
We will use music with our infant even without research *	1.6 ± 0.27	1.2 ± 0.86	0.27
We do not know how to use music in the NICU *	3.2 ± 0.46	3.1 ± 0.3	0.47
Music will enhance bonding with my baby *	4.2 ± 0.9	2.7 ± 1.3	0.02

Annotation: * Mean ± Standard Deviation. For the answer categories: (1) Strongly disagree, (2) Disagree, (3) Neither agree nor disagree, (4) Agree, (5) Strongly agree.

**Table 3:** Characteristics of those who consented or declined to participate in music intervention studies.

Characteristic	Consented (*n* = 116)	Declined (*n* = 87)	*p*-Value
Maternal age (years) *	28.6 ± 5.7	25.3 ± 3.2	0.24
Single-parent family, *n* (%)	15 (13)	20 (22)	0.05
Maternal education (years) *	14 ± 2.9	12 ± 3.1	0.19
Paternal education (years) *	12 ± 3.2	14 ± 2.1	0.33
Employed, *n* (%)	87 (75)	62 (78)	0.24
Gestational age (weeks) *	28.1 ± 2.5	27.6 ± 2.3	0.36
Birth weight (grams) *	1057 ± 186	859 ± 147	0.12
Ethnic origin, *n* (%)			
Jewish	59 (50)	45 (52)	0.16
Arab	41 (35)	38 (43)	
Other	16 (15)	4 (5)	
Religious, *n* (%)	41 (35)	34 (39)	0.09
Infant male sex, *n* (%)	53 (46)	43 (49)	0.33
Singleton, *n* (%)	104 (90)	80 (92)	0.24
Previous parental research involvement, *n* (%)	17 (15)	10 (11)	0.17
Post-menstrual age at consent/decline, (weeks) *	30.6 ± 1.9	31.3 ± 2.4	0.06
Age at consent/decline (days) *	17 ± 2.3	28 ± 4.7	0.03
Clinical Risk Index for Babies score *	6.1 ± 2.7	3.68 ± 4.1	0.04

Annotation: * Mean ± Standard Deviation.

## Data Availability

The data presented in this study are available on request from the corresponding author. The data are not publicly available due to IRB regulations.
